# Measuring land use impacts of national dietary guidelines: a scoping review of methods, trade-offs and policy implications

**DOI:** 10.1088/2976-601X/ae7089

**Published:** 2026-07-28

**Authors:** Niamh M Kelly, Viola King Forbes, Rebecca Wells, Richard Pearson, Kelly Parsons, Christian Reynolds

**Affiliations:** 1 Centre for Food Policy, City St George’s University of London, London, United Kingdom; 2 Centre for Biodiversity and Environment Research, University College London, London, United Kingdom; 3 Business School, University of Sussex, Brighton, United Kingdom; 4 National Resources Institute, University of Greenwich, Kent, United Kingdom; 5 MRC Epidemiology Unit, School of Clinical Medicine, University of Cambridge, Cambridge, United Kingdom

**Keywords:** healthy diets, food policy, food systems, land systems, sustainable diets

## Abstract

*Background.* As agriculture uses a substantial proportion of the world’s habitable land, it is important to consider how the food we eat, and different dietary patterns, impact the pressures put on land. *Aim.* This scoping review aims to 1. explore the approaches to measuring potential land use impacts of aligning diets or the food supply with national dietary guidelines, 2. summarise the additional environmental factors included in these studies, and specifically how often biodiversity is included, 3. identify policy recommendations made. *Method.* The Joanna Briggs Institute scoping review framework was used to identify studies measuring potential land use changes of shifting national diets or the food supply towards national dietary guidelines. Only official national dietary guidelines were included. Due to heterogeneity in how dietary and environmental impacts were calculated, results are represented in terms of increases and decreases, rather than average percentage change. *Results.* Of the forty-five papers included, only 4 measured biodiversity impacts, compared to 35 measuring greenhouse gas (GHG) emissions, 24 water, and 10 fertiliser use. Most studies used life cycle assessment to measure the potential land use and environmental impacts. All studies found an increase in fruit and vegetables would be needed to meet dietary guidelines, and all but two found a reduction in meat is required. Papers mostly reported reduction in resource in land use and GHGs (28/45 and 22/35 papers, respectively). Impacts on water, energy and biodiversity, which were less frequently included, were more mixed. Over half of the papers included policy recommendations (*n* = 29), with the most being to incorporate sustainability into dietary guidelines. *Conclusion.* A wider range of environmental outcomes should be included when assessing the environmental sustainability of dietary changes to get a more holistic view of the potential trade-offs and co-benefits that need to be navigated with these dietary shifts.

## 1. Introduction

Land is facing increasing demands from multiple sectors including nature conservation, housing and infrastructure, and particularly from food production. Almost half (44%) of the world’s habitable land is used for agriculture (Ritchie and Roser 2019). What we eat has a major impact on the environmental resources. Land use requirements vary by what and how food is being produced. Globally, two thirds of agricultural land is used for grazing animals (FAO 2025) and approximately one third of cropland is used to grow animal feed (Poore and Nemecek 2018, Ritchie and Roser 2019, FAO 2025). The type of food and method of production can also impact the land quality and soil health, with overgrazing and intensification contributing to soil degradation. Due to these interconnections, dietary change is often encouraged to reduce the environmental burden of agriculture.

While the types of dietary change needed for healthy diets varies across contexts, national dietary guidelines are a tool to advise the public on healthy eating, tailored to national contexts, and are currently present in over 100 countries (FAO 2024). These guidelines usually consist of recommendations for portions of overarching food groups such as fruit and vegetables, cereals or grains, potatoes and other starchy carbohydrates, dairy products, protein sources which can be subdivided into meat, fish, eggs and beans, peas and pulses, oils and sometimes discretionary foods high in fat, salt and sugar. Details of these food groups and recommended servings are tailored to the diets and culture of a nation. This makes national dietary guidelines more country-specific compared to broader dietary recommendations from international organisations.

It is often suggested that healthier diets are also more environmentally sustainable, and there is growing interest in understanding the sustainability trade-offs and co-benefits of eating healthy diets (Springmann *et al* 2020, Webb *et al* 2023). The outcome of sustainability can differ based on what environmental factors are included. (Springmann *et al* 2020, Webb *et al* 2023). Land use and greenhouse gas emissions (GHGs) are commonly included in sustainability assessments of diets. However, as we become more aware of how trade-offs and co-benefits can vary between different environmental factors in the shift towards healthy diets, it is important that we expand the range of environmental impacts that are analysed and also include factors such as biodiversity. Food systems are both dependent on biodiversity, particularly pollinators which are needed for 75% of crops (Our World in Data 2021), while also being the main driver of biodiversity loss on land. Destruction of habitats linked with land use change, monocropping, and the use of chemicals such as pesticides, insecticides, and fertilisers are all impacting levels of biodiversity (Benton *et al* 2021). Biodiversity is increasingly on the agenda for food systems transformation, yet is often not included in analyses of environmental impacts of dietary change, compared to other environmental factors.

A range of approaches can be used to measure the environmental impacts of individual foods and dietary patterns. When measuring potential land use changes, this can be done spatially, by looking at where land use changes could physical happen, or through methods such as life cycle analysis, which takes into account the environmental impacts of the production of foods at each step of the food supply chain (‘ISO 14 044,’ 2006, p. 4). In order to develop a more standardised approach to exploring these potential impacts current inconsistencies and gaps must be identified and addressed.

The aim of this scoping review is threefold. 1. to explore the range of approaches and data used to model the potential land use impacts of shifting diets or food supply to align with national dietary guidelines; 2. to look at what other environmental factors are included in these studies in addition to land use, and specifically how often biodiversity is included, if at all; 3. identify policy recommendations made by authors. This scoping review focuses on national dietary guidelines as we are interested in the environmental sustainability of current national recommendations and how this compares to current average diets within a country. To broaden the scope of this review, we consider both current average diets and the food supply, which is the total food available for consumption in a country, as both measures are frequently used in this type of analysis.

The growing interest in this area is evident from the publication of reviews about sustainable healthy diets (Steenson and Buttriss 2021, Webb *et al* 2023, Niu *et al* 2024, Goossens and Schmidt 2025). Webb and colleagues (2023) explored 42 studies which assessed the impacts of at least one dietary pattern (which could include, for example, current diets, the EAT-Lancet diet, Mediterranean diet, flexitarian or vegan diet) on at least two pillars of sustainability (health, social, environmental, economic). The authors found that environmental outcomes were the most frequently analysed outcome, but engagement with policy was lacking in papers included in the review (Webb *et al* 2023). Policy implications of results are important to consider, as policies can both impact how land is used as well as influence a population’s diet. Meanwhile, Steenson and colleagues carried out a narrative review of 29 studies from high-income countries which used different approaches to define healthy and sustainable dietary patterns (Steenson and Buttriss 2021). While these previous reviews focus on overall healthy diets, this current review differs by focusing specifically on the impact of alignment with national dietary guidelines, as these are usually the diets endorsed by national governments and have the potential to be used across national policy for a coherent approach to dietary change. This current review also explores the methods used to measure potential environmental impacts of the dietary shifts, the specific environmental factors included, as well as the policy recommendations put forward by authors.

## 2. Methods

This scoping review was carried out following the Joanna Briggs Institute (JBI) approach (Aromataris *et al* 2024), using the Population, Concept, Context (PCC) framework to develop the search strategy and inclusion/exclusion criteria. The PCC for the current review is summarised in table 1, and detailed inclusion criteria can be found in the supplementary material. The initial search was carried out in February 2024, and re-run in November 2024 to identify any new publications. At this point all titles and abstracts found during the search were double screened by NK and VKF. This process was carried out following an *a priori* protocol (available at https://doi.org/10.6084/m9.figshare.30258223).

**Table 1.** Population, Concept and Context for current review.

Population	All countries with national dietary guidelines
Concept	Measuring the potential land use impacts of aligning diets with national dietary guidelines
Context	Impacts can be measured on a local, national or international scale, provided the national dietary guidelines form the basis of one scenario, rather than global dietary guidelines

### 2.1. Searching for the evidence

The search strategy in table 2 was developed through an iterative process, to identify papers measuring the potential land use impacts of aligning diets or the food supply with national dietary guidelines. Search terms were changed to only include words specifically linked with dietary guidelines, rather than general healthy diets, or specific diets such as the Mediterranean or EAT-Lancet diet. No restrictions were placed on publication date.

**Table 2.** Final search strategy.

Search strategy
‘dietary guidelines’ OR ‘recommended diet*’ OR ‘food pyramid’ OR ‘healthy eating guidelines’ AND ‘Land use’ OR cropland AND model* OR scenario* OR simulat*

### 2.2. Selecting the evidence

Scopus, Food Science and Web of Science were searched using the strategy above, and results were filtered to peer reviewed articles. This resulted in 1,481 papers, which were uploaded onto Rayyan Review Management Software, and 191 duplicates were identified by the software and removed (Ouzzani *et al* 2016). Titles and abstracts of the remaining papers were screened by NK and VKF, according to the inclusion and exclusion criteria listed in Table A1 in the appendix. When it was unclear from the abstract whether a paper met all inclusion criteria, the paper was included in full text screening for clarification. After this initial screening, 158 papers were included for full text screening. All full texts were screened by NK, and a subset of 20% were screened by VKF. Both researchers met to compare and validate the decisions. As the decisions to include/exclude papers aligned between both researchers, NK completed the final 80% of screening independently.

### 2.3. Extracting the evidence

**Figure 1.**
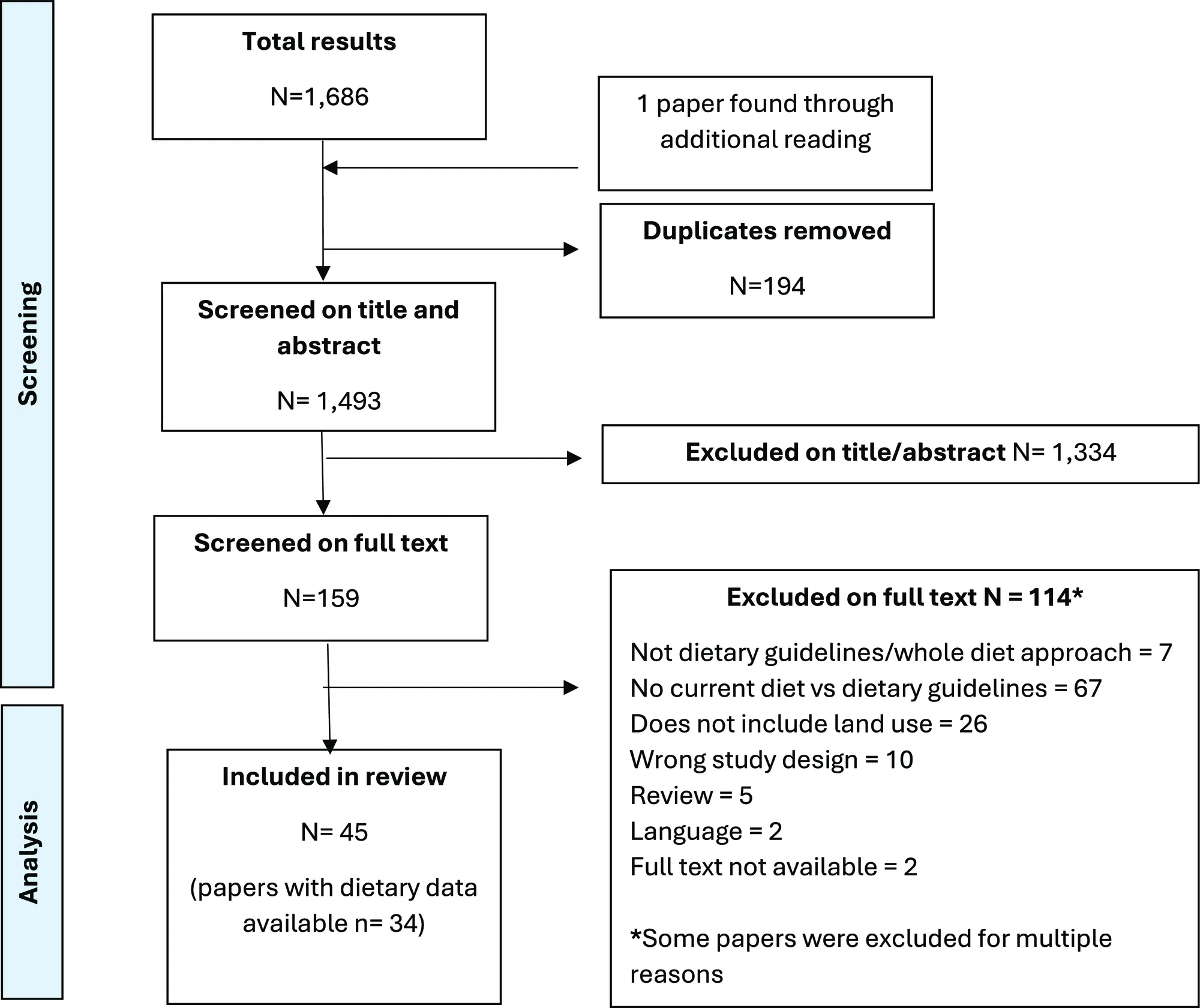
PRISMA flow chart for scoping review.

A final set of 45 papers were included in this review, see PRISMA chart in figure 1 (PRISMA 2020). The full list of all papers can be found in the supplementary material. The following data was captured in a spreadsheet: country, year of publication, method, data sources (diet and land), impacts on diet, land, any additional environmental factors, cost and health, and policy recommendations made by authors. These data points were selected and adapted based on the JBI guidance for scoping review data extraction (Aromataris *et al* 2024). Data was first extracted from a subset of 10 papers, and any necessary alterations were made to the data extraction framework. Figures were created with Datawrapper (DatawrapperGmbH 2024).

There was heterogeneity in how studies reported changes in dietary intakes, with some reporting in g/person/day, servings/day, or kcal/person/day, while others reported mega tonnes of food per year. Therefore, the percentage change for each food group was calculated for this review, to allow for better comparability. Results for diet change were grouped together into fruit and vegetables, starchy carbohydrates, meat, fish, dairy, sugar and oils, and the percentage change was calculated. If the exact data for changes in diet and environmental factors was not available in the paper or supplementary materials, the authors were contacted and asked for the underlying data. If there was no response from an author, one follow-up email was sent, and, if there was still no response the paper was not included in the analysis of dietary impacts but was still included in the analysis of typologies of papers.

#### 2.3.1. Studies with multiple scenarios

When a study included multiple scenarios complying with dietary guidelines, the diet considered closest to current diets was used as the comparison to baseline diets. For example, when a paper had multiple diets that all aligned with dietary guidelines and one was an omnivorous/mixed diet, and other scenarios were vegetarian, vegan etc, the omnivorous diet was taken as the comparison. This was to ensure the comparison was as relevant as possible to the general public, and because while many dietary guidelines may encourage reductions in animal products, the recommendations in national dietary guidelines are not usually advocating for a strictly vegan or vegetarian diet. Similarly, when studies included different levels of compliance, e.g. 80%, 90% and 100%, 100% compliance was used as the comparison to allow for better comparison with the other analyses in this review which only include full compliance with national dietary guidelines in their scenarios. The same principle was applied when diets were modelled at different time intervals e.g. 2020, 2030, 2050; the time period closest to the baseline diet was selected (usually 2020 or 2010). This was to minimise the impacts that other factors such as population or climate change could have on the results, as the data of interest were the impacts of diet change specifically. When analysis was carried out on a global scale, while also reporting results by country or region, the global level data were included in the analysis for this review.

In line with this thinking, when researchers included alternative scenarios of reduced food waste or changes in income levels, when possible, the dietary scenarios without these additional changes were used, to focus solely on the impacts of dietary shift towards dietary guidelines. When it was not possible to select scenarios without additional changes other than diet, these changes were noted in a table, a link to which can be found in supplementary material A2.

#### 2.3.2. Optimisation studies

As many of the optimisation studies had additional constraints, these were allowed as long as it was determined that these additional constraints would not alter the composition of the diet in a way that would make it incomparable with other papers. For example, some optimisation studies minimised the changes needed to current diets, while still complying with national dietary guidelines, to improve the acceptability of the diet. Similarly, when allocating changes to land use, some studies optimised the allocation of land use to maximise efficiency in water use, or reduce impacts of climate. These additional constraints were accepted.

#### 2.3.3. Policy recommendations

A policy recommendation was identified in the discussion or conclusion section of a paper when authors suggested actions such as policies, taxes, regulations, reforms ‘should’ be implemented by policymakers, or that ‘there is a need’ to change policies or actions. Some authors discuss policy more broadly, for example ‘We are unlikely to achieve significant diet shifts without either gradual cultural changes or potentially unpopular measures such as taxing meat, either directly through an excise or sales tax on purchases, or indirectly via a carbon tax’ (p5) (Read *et al* 2022). This was not deemed a direct policy recommendation as it was more a discussion of policy options.

## 3. Results

**Figure 2.**
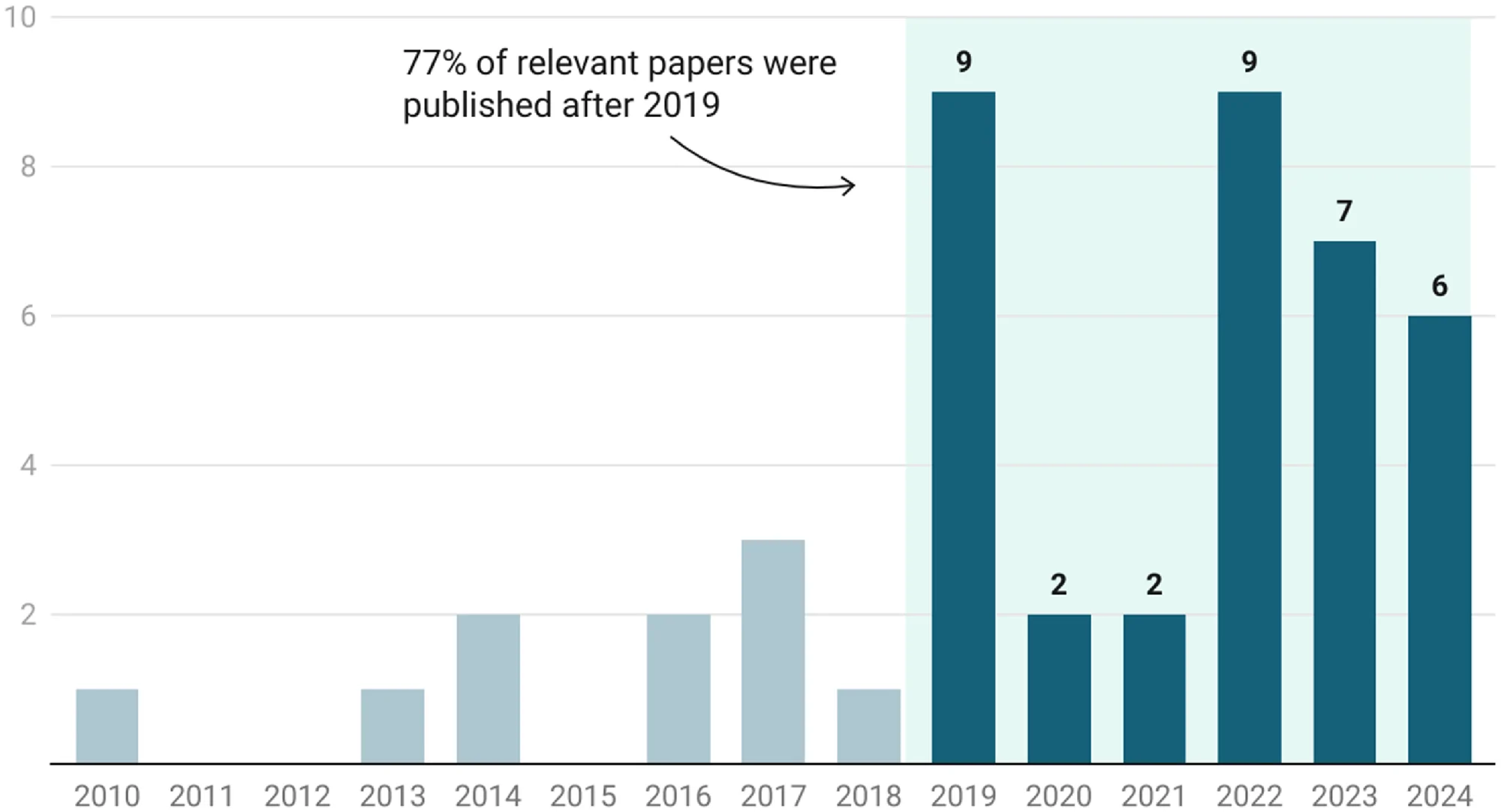
Number of papers published by year.

**Figure 3.**
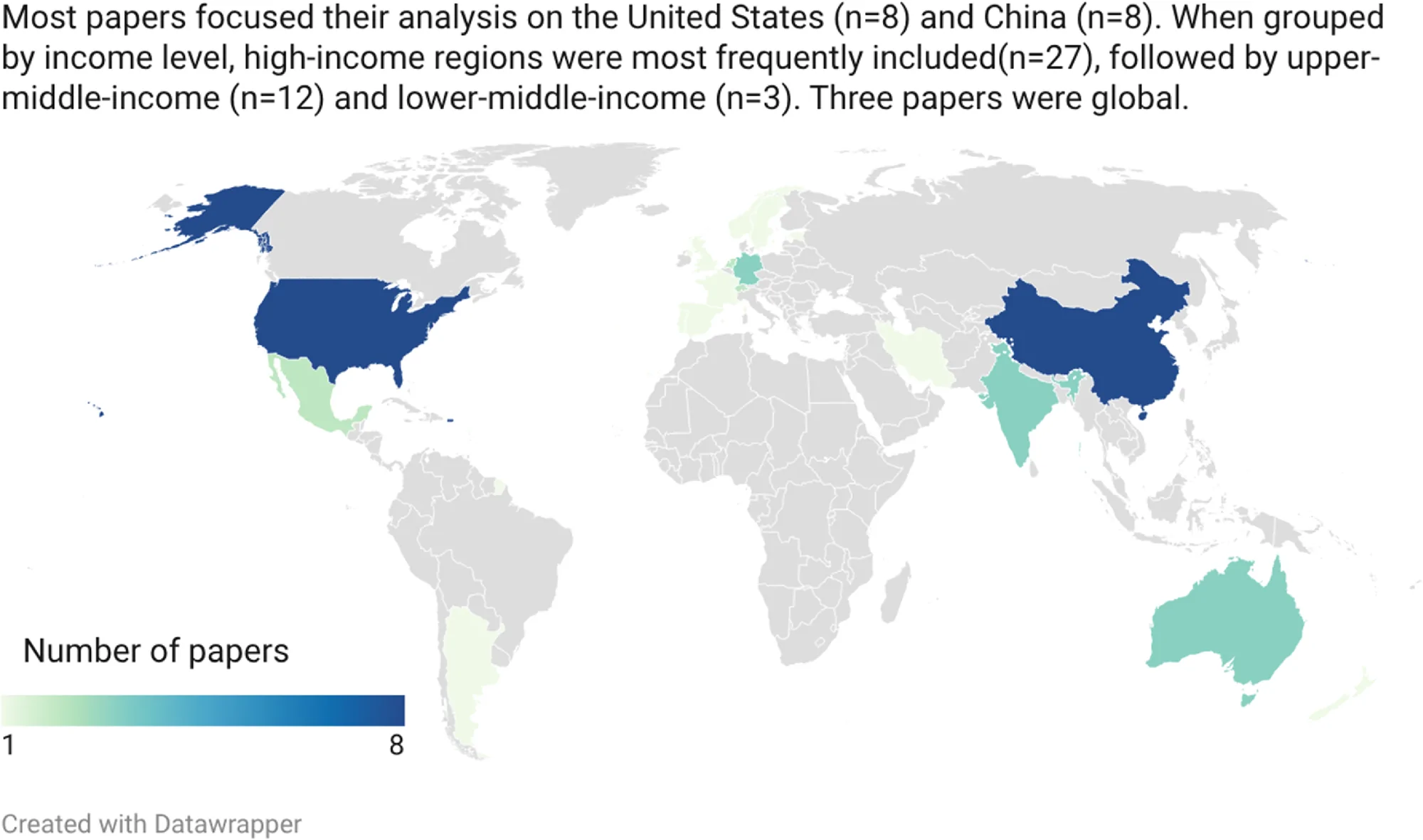
Distribution of countries covered by the papers included in this review.

In total, 45 papers were included in the review, the majority of which (*n* = 34, 77%) were published since 2019 (see figure 2). The geographic focus of the papers mostly clustered around the United States (*n*= 8) and China (*n* = 8), (see figure 3). However, when grouped into regions Europe had the most papers (*n* = 15), followed by North America (*n* = 10) and Asia (*n* = 11).

### 3.1. Approaches used to measure potential land use change

**Figure 4.**
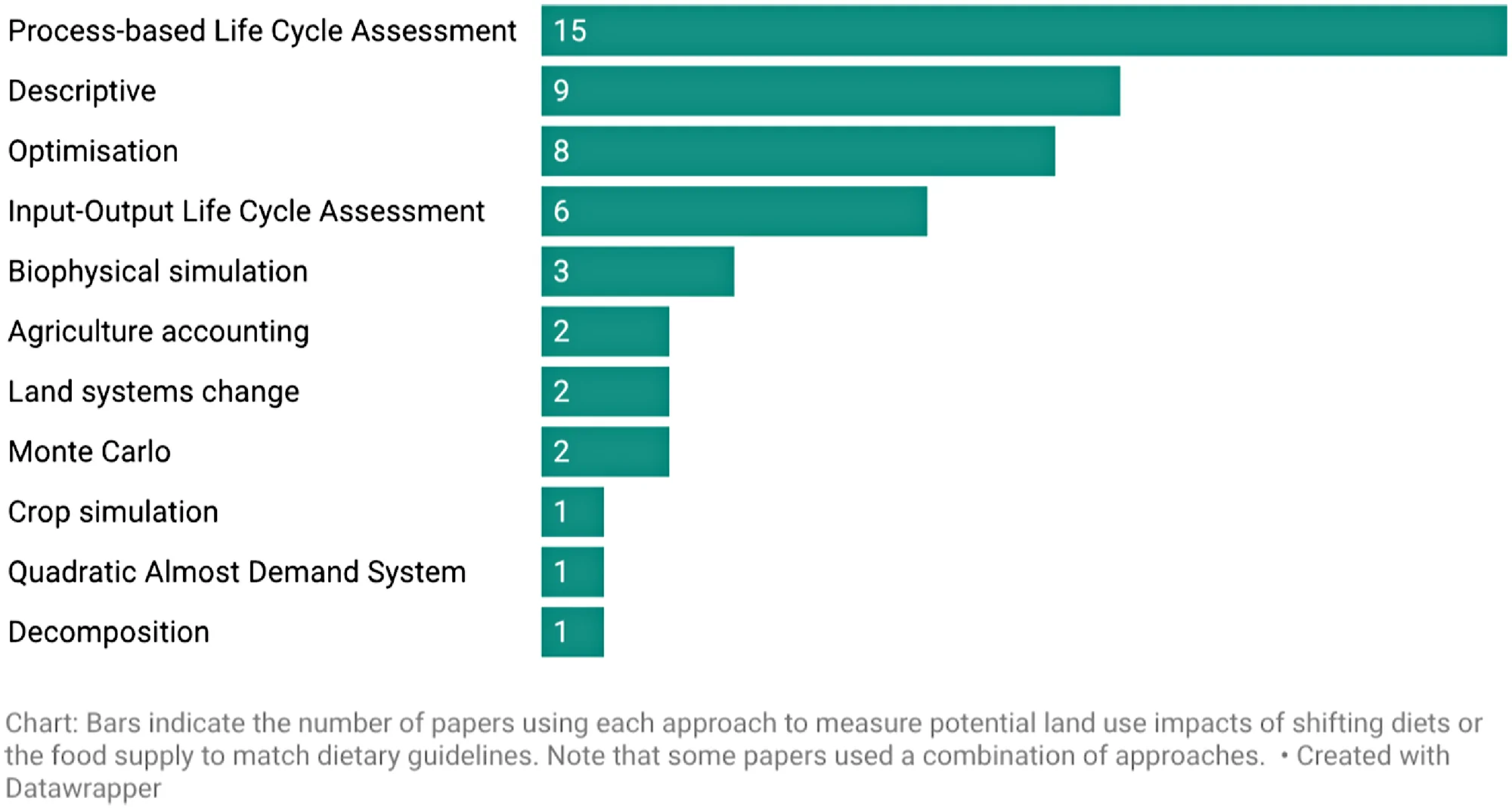
Approaches used to measure land impacts of dietary scenarios.

Most papers used either a life cycle analysis (LCA) or optimisation to assess the environmental impacts of dietary shifts (figure 4). LCA was most common, being used in a total of 20 papers (see Table A3 in the supplementary material), including two papers which also used optimisation to create dietary scenarios (van Dooren and Aiking 2016, Muñoz-Martínez *et al* 2023). Of the papers that used LCA, fifteen were process-based and six took an input–output approach. Eight studies used an optimisation approach, where they created an optimised diet which would meet the national dietary guidelines, with some studies including additional constraints around acceptability, and environmental factors. Some US studies also used the Foodprint model (Peters *et al* 2016, Conrad *et al* 2018), a national biophysical simulation model which factors in dual cropping, where multiple crops are grown on the same area of land at different times during the year (Conrad *et al* 2018). Table A3 in the supplementary material shows an overview of which approaches were used by which papers.

Even when using a similar method, there was a diversity in the approaches taken by authors. The range of system boundaries used in the papers are shown in Table A.4. in the supplementary material. The most common boundary, set by four papers was ‘cradle-to-gate’, followed by ‘cradle-to-store’, used by three. Cradle-to- ‘plate’, ‘mouth’ and ‘grave’ and ‘farm-to-fork’ were set as the LCA boundary by one paper each. This makes it difficult to ascertain exactly what is included, particularly when different terms are used for the same stage. Because exact definitions of system boundaries were not provided in papers, the assumption made in this study is that these terms (plate, mouth, grave and fork) all relate to the final stage of the life cycle of food

### 3.2. Data sources

#### 3.2.1. Diet

A range of datasets were used for establishing the baseline diet. National Nutrition Surveys were the most common data source (36), followed by FAOSTAT (*n* = 13) shown in Table A5 in the supplementary material. These figures include three papers which used both sources. The US Economic Research Service (ERS) Food Loss and Waste Adjusted data was also a common data source used in the US-based studies (*n* = 3). Analyses focused on the consumption of foods more often than the food supply. Some studies using national dietary surveys had quite a large period between when the original data was collected, and when the paper was published (see figure S1 in supplementary material). For example, for the research carried out by van Dooren *et al* (2014), a national dietary survey from 1998 was used; a 16 year gap. For most other studies using national datasets, the gap was close to 5–10 years. While Food Balance Sheets from FAOSTAT are produced each year, and therefore papers using these datasets could make use of more up-to-date data, many papers used FAOSTAT data from 2010 to match the timeline of the environmental data used (Springmann *et al* 2020), or due to their analysis tool being based on 2010 dietary data (Wu *et al* 2023). Furthermore, as there is often a two-year delay in FAOSTAT food balance sheet data becoming available, and there was a change in methodology after 2011, there may have been an issue with accessing more recent data at the time some analysis was carried out.

#### 3.2.2. Land use

A combination of data sources was used to determine land use changes, including FAO data, results from previous studies, or national land data. Previous studies were the most common data source (*n* = 18), followed by FAOSTAT (*n* = 17), and national datasets (*n* = 12). Some papers used more than one data source, and the detailed breakdown of types of data sources for each paper can be found in Table A5. Just two papers, Huang *et al* and Read *et al*, used spatial datasets (Huang *et al* 2019, Read *et al* 2022). Of these two, only Read and colleagues included analysis on biodiversity, which will be discussed in more detail later on in this results section.

### 3.3. Impacts of aligning diets with national dietary guidelines

#### 3.3.1. Dietary impacts

**Figure 5.**
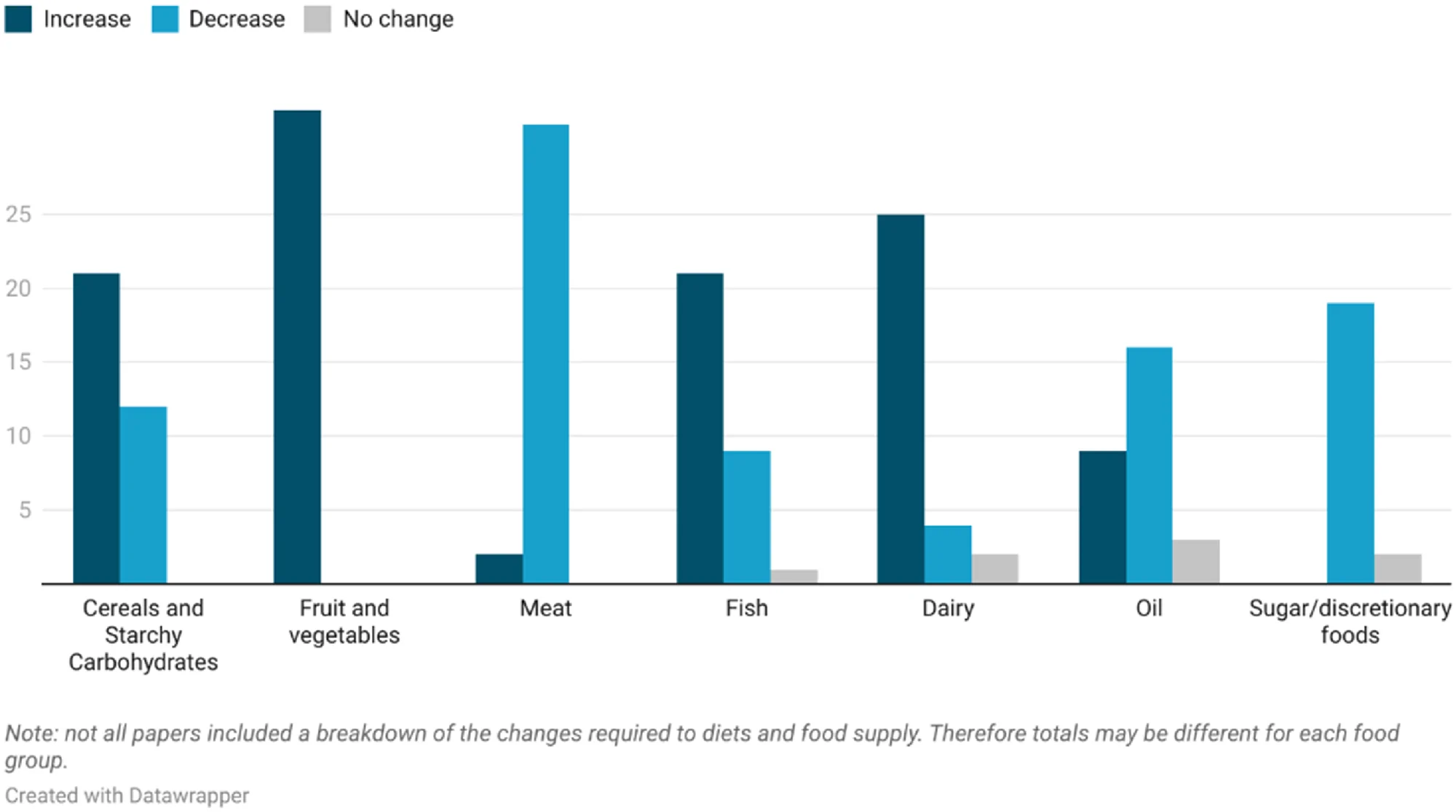
Number of papers identifying an increase or decrease in food groups when shifting diets/food supply with national dietary guidelines.

As the approach for reporting changes in diet or food supply varied from study to study, the percentage change was calculated for each paper to allow for better comparability (figure 5). The full detailed summary of the dietary and environmental impacts can be found in the supplementary material, along with a breakdown of the dietary results for Asia, Europe and North America (figure S2). There were insufficient numbers of papers with dietary data available to summarise other regions. An overview of the main findings across all papers is summarised below.

In most papers the scenario for aligning diets with dietary guidelines involved reducing meat (−7% to −74%) and sugar (−3% to −100%) and increasing fruit and vegetables (+17 to +207%), compared to baseline diets. (Ridoutt *et al* 2020, 2021), which focused on Australia, were the only papers which found there would need to be an increase in meat (20.59% and 20.89%, respectively) (Ridoutt *et al* 2020, 2021). This contrasts with (Candy *et al* 2019), also based on Australia, which found a reduction in the consumption of meat in their “Healthy-Mixed“ (dietary guidelines) scenario (exact percentages were not possible to calculate due to lack of available data, however the reduction can be seen in figure 1(a) in the referenced paper). The dietary scenarios in (Candy *et al* 2019), had additional requirements (reducing the consumption of meat, and including more plant-based foods), which may have contributed to these differences in results (Candy *et al* 2019). Other potential reasoning for this outlier of increased meat consumption from Ridoutt *et al*, 2021, 2020 is explored further in the discussion.

Results for dairy were more mixed with five papers identifying a reduction needed in dairy, which were based on diets in England and Wales, and Germany (Arnoult *et al* 2010, Chan *et al* 2022). Two papers indicated no change in dairy (Peters *et al* 2016, Ibarrola-Rivas *et al* 2022), while all other papers which included dairy indicated an increase, some up to 500%. The papers with the highest level of increases for dairy were based on China (He *et al* 2019, Zhu *et al* 2023a), followed by Argentina with an increase of 235% (Arrieta *et al* 2022).

#### 3.3.2. Land impacts

The impact of diet change on land use was mostly represented as the change in total land used for agriculture. Some papers used measures such as cropland-scarcity which takes into account both the area of land use and the productive capability of the land used (Ridoutt *et al* 2021), or carrying capacity of the land which identifies how many people can be fed from the available agricultural land in a dietary scenario (Peters *et al* 2016) and land use scores (van Dooren *et al* 2014). Due to this variation in measurement of land use change, results from different studies were not always directly comparable. The changes in land use were mostly represented as a total change across all land types. Two authors broke down the impact based on cropland and land for grazing, and recommended that future results should be presented separately for cropland and livestock, as the impacts are likely to be different (Peters *et al* 2016, Ibarrola-Rivas *et al* 2022).

Of the papers that measured changes in the amount of land used, twenty-eight papers found a reduction in land use, ranging from 1–32%, which is beneficial for reducing resource use. These reductions likely came mostly from the reductions in meat. Fifteen papers found increases in land use, ranging from 1.9–86.9%. Geographically, these studies focused on China, India, Iran, Argentina, New Zealand and the US and there was one global study. The largest increase found in land use was by Zhu and colleagues (2023b), with an increase of 86.9%.

#### 3.3.3. Other environmental outcomes

Nine papers did not include any other environmental impacts in addition to land use. GHG emissions were the most common additional environmental factor included in analysis of the impacts of the dietary scenarios measured in 35 papers, followed by water use (*n* = 24), fertiliser (*n* = 9) and energy use (*n* = 8). Biodiversity was one of the least common outcomes included, featuring in only four papers, the same as eutrophication.

**Figure 6.**
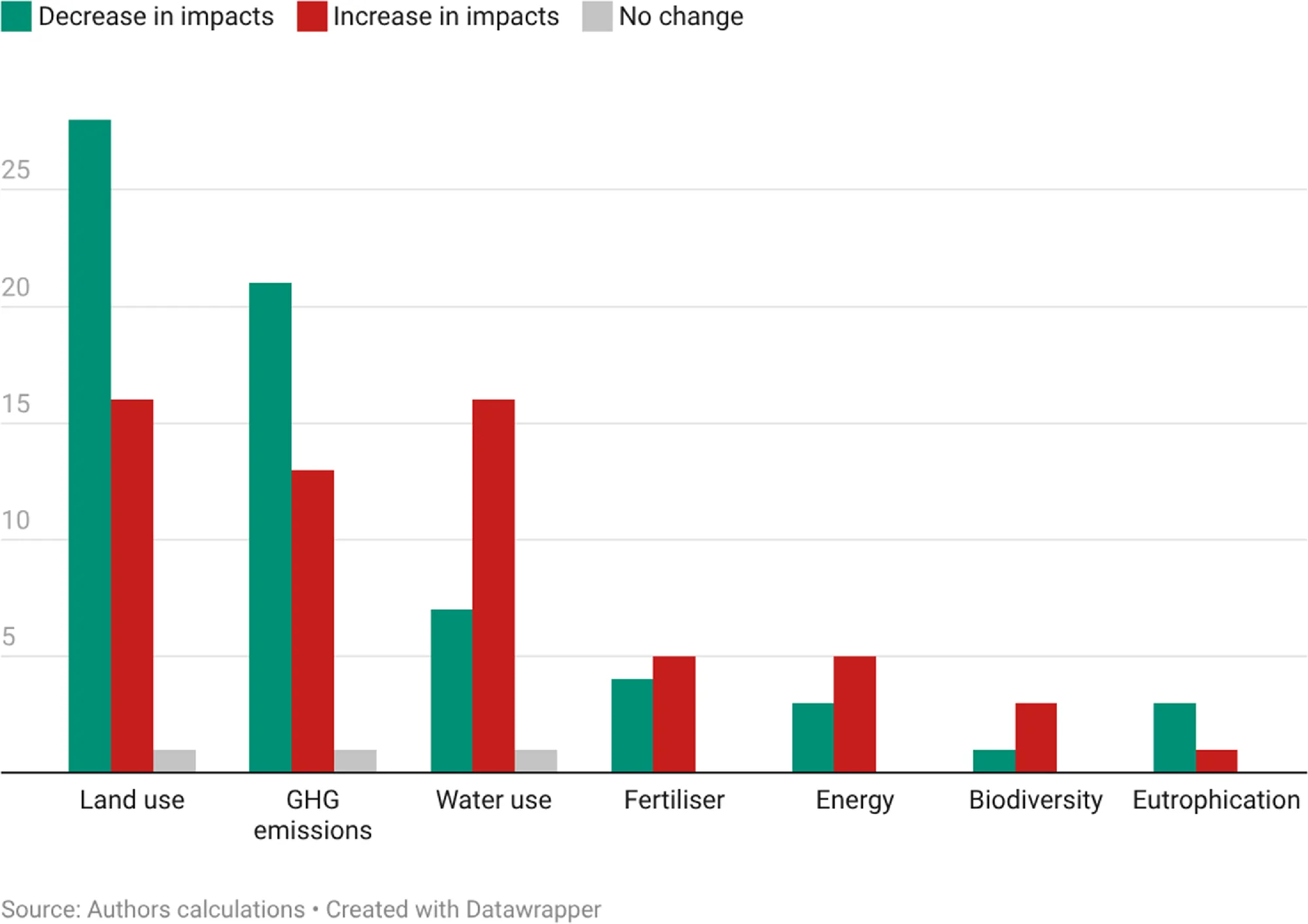
Number of papers indicating an increase or decrease in environmental impacts when shifting diets/food supply with national dietary guidelines.

As with the dietary changes, environmental impacts were summarised in figure 6 as increases and decreases rather than exact percentage changes due to the variation in approaches used (i.e. optimisation study versus LCA), and the different LCA boundaries used (e.g. cradle-to-gate versus cradle-to-grave). Of the 35 papers measuring impacts of GHG emissions, 13 found an increase in emissions, ranging from 3% to 10%, while 21 papers found a decrease in emissions from −11% to −54% (a full breakdown of these results and references is included in the supplementary material A4). Over three quarters of papers which measured GHGs emissions reported results as CO_2_ equivalent (*n* = 28). Of these, three papers specify that that CO_2_, CH_4_, and N_2_O were included, while one paper stated that only CH_4_ and N_2_O were included in their analysis as the CO_2_ was mainly linked to non-agricultural industries (Springmann *et al* 2020). One paper reported the results as global warming potential, which is the ability of a GHG to trap heat over a set period of time (for example 100 years) in comparison to CO_2_ (Coelho *et al* 2016), and one paper looked at the change in Sustainable Development Goal (SDG) indicator score for emissions (Change in SDG indicator 13.2.2–0.01% ± 11%) (Liu *et al* 2022). Changes in water use ranged from a reduction of 26.1% to an increase of 200%. Of the 24 papers that measure impacts on water use, 16 found an increase in water use. It may be helpful In future to distinguish between blue water (in rivers, lake, groundwater reserves) and green water (water sources such as rainfall available in the soil for plants) use, as Birney found blue water use would increase by 15%, while green water use would reduce by 7% (Birney *et al* 2017).

#### 3.3.4. Biodiversity

As noted above, only four out of 45 papers included assessments of the impacts on biodiversity (Martin and Brandão 2017, Ridoutt *et al* 2020, Springmann *et al* 2020, Read *et al* 2022). These assessments mostly used proxy measures, such as ‘biodiversity damage potential’ (Martin and Brandão 2017) or ‘threats to biodiversity’ (Read *et al* 2022), or the compatibility of national dietary guidelines with the Aichi biodiversity target, set by the United Nations’ Convention on Biological Diversity, on limiting land use change (Springmann *et al* 2020). Threats to biodiversity were calculated by multiplying land use by biodiversity metrics that characterise the number of potential global species extinctions in taxonomic groups per square meter of natural land converted to agricultural land (Read *et al* 2022). The impacts on these biodiversity measures were mixed, with one study finding a reduction in threats to biodiversity (Martin and Brandão 2017), and another finding an increase in threats to biodiversity, which authors suggested may be due to the increase in imports of fruit from tropical countries (Read *et al* 2022). Another paper measured the potential impacts on biotic natural resource depletion, but highlighted in their limitations that impacts on biodiversity could not be measured due to the lack of spatial data (Coelho *et al* 2016).

Of the papers that did not include biodiversity in their analysis, 22 mentioned biodiversity in some way in their paper. Only two papers included a reason why biodiversity was not included in their analysis and that was due to lack of data (Aleksandrowicz *et al* 2019, Chen *et al* 2019). Three papers either highlighted the absence of biodiversity analysis as a limitation, or suggested that biodiversity should be included in future research (Chaudhary and Krishna 2021, Chan *et al* 2022, Frehner *et al* 2022). Some papers identified land use (and eutrophication (van Dooren *et al* 2014)) as good proxies for biodiversity impacts (van Dooren *et al* 2014, van Dooren and Aiking 2016), while others referred to the potential positive biodiversity impacts of the land use changes modelled in their analysis. Although most papers did not necessarily recognise the absence of biodiversity assessment as a limitation, or identify it as an area for future researchers, they did mention the importance of biodiversity for food systems, or the impact of land use change on biodiversity in their introduction or discussion.

#### 3.3.5. Cost

When looking at the impact of dietary change on costs, six papers reported the impact dietary change would have on the cost of diets itself, while one reported impacts on labour costs. Of those which looked at the cost of the dietary guidelines compared to current diets, three found that the cost of diets would decrease by 27%, 35% and 21%, respectively (Chen *et al* 2019, Frehner *et al* 2022, Unar-Munguía *et al* 2024). On the other hand, Arrieta and colleagues found that the dietary guidelines diet would be the most expensive compared to no ruminant-meat, EAT-Lancet or vegan diets, and plant-based diets were least-expensive (Arrieta *et al* 2022), while Hu found the cost of dietary guidelines was almost double the current diets, with a 70% increase (Hu *et al* 2022). Muñoz-Martínez and colleagues found an increase of 15.14% (Muñoz-Martínez *et al* 2023). Arnoult and colleagues included analysis of the changes to the cost of labour, and found that under the dietary guideline scenario, the cost of labour would decrease by 9.1% due to reduced livestock farming, and the net margin of a farm would increase 143.9% (excluding Pillar I payments from European Union Common Agricultural Policy, and Agri-environment Schemes, which were in place at the time) (Arnoult *et al* 2010). The authors do not analyse how these changes in costs could transfer to consumers, if at all.

Changes in the cost of diets were not always evenly spread. However, as only seven papers in this review included cost analysis, and not all examined impacts across income groups, there were too few studies to ascertain meaningful patterns across different income groups. In Mexico, under a healthy diet scenario costs would reduce more in urban areas compared to rural (Unar-Munguía *et al* 2024). Hu and colleagues also found that households living in rural China would face a steeper increase in the cost of a healthy diet than those in more urban regions (Hu *et al* 2022). When looking at the impacts of dietary change on labour costs, Arnoult and colleagues suggested that these impacts are likely to be felt unevenly across England and Wales, as the East of England is likely to benefit most from increase in horticulture, and the West of Wales would be most impacted by the reduction in livestock farming, and therefore loss of labour in the area (Arnoult *et al* 2010).

### 3.4. Policy recommendations

Twenty-nine papers included direct policy recommendations. The main themes of these recommendations were: incorporate environmental sustainability into dietary guidelines (*n* = 12), having a holistic and coherent food systems approach (*n* = 7), the need for a combined supply and demand side approach (*n* = 5), implement measures to encourage dietary change (*n* = 5), consider socioeconomic factors of food-environment nexus and related policies (*n* = 5), implement policies to change food production and promote sustainability (*n* = 5), use regional approaches to develop food policy (*n* = 3), and foster transdisciplinary collaborations (*n* = 2). Note that some papers included more than one recommendation. More detailed descriptions of specific policy measures suggested in these papers are summarised in table 3.

**Table 3.** Summary of the main policy recommendations from authors.

Actionable Policy recommendation	References
**Incorporate environmental sustainability into dietary guidelines:** update dietary guidelines with environmental sustainability criteria, reduce the amount of meat in dietary guidelines and promote more plant-based diets.	(Arrieta *et al* 2022; Ibarrola-Rivas *et al* 2022; Jennings *et al* 2023; Li *et al* 2024; Martin and Brandão 2017; Scherer *et al* 2019; Sobhani S R *et al* 2022; Springmann *et al* 2020; van Dooren *et al* 2014; Wood *et al* 2019; Yin *et al* 2020; Zhu *et al* 2023b),

**Develop holistic and coherent food systems approach:** use a ‘multi-pronged approach’ such as developing a framework that recognises interlinked elements of the food system, coordinated action plans, support dietary guidelines with broader food systems policies to protect security of food supply, and ensure policies from other government departments do not contradict dietary guidelines.	(Arrieta *et al* 2022; Candy *et al* 2019; Frehner *et al* 2022; Lengle *et al* 2024; Meier T *et al* 2014; Springmann *et al* 2020; Wood *et al* 2019)

**Combine supply and demand approach for sustainable production and consumption:** support production of more legumes, encourage consumers to decrease consumption of animal protein (e.g. through taxing meat) and increase plant-based proteins, and reduce food loss and waste.	(Aleksandrowicz *et al* 2019; Chan *et al* 2022; Chaudhary and Krishna 2021; Li *et al* 2024; Meier *et al* 2014)

**Implement measures to encourage dietary change:** increase awareness of healthy diets, and support health education. Change food environments to offer healthier and sustainable options.	(Bashiri *et al* 2024, Huang *et al* 2019; Lengle *et al* 2024; Martin and Brandão 2017; Scherer *et al* 2019)

**Consider socioeconomic factors of food-environment nexus and related policies:** reduce barriers to sustainable and healthy diets using context-based strategies that take socioeconomic factors into account, and consider the socio-political situation when developing initiatives to build resilient food systems. Improve pension systems to support aging population to afford healthy diets.	(He *et al* 2019; Hu *et al* 2022; Li *et al* 2024; Muñoz-Martínez *et al* 2023; Stone *et al* 2023),

**Implement policies to change food production and promote sustainability:** include food waste in animal feed, reduce economic gains for sugar and cereal crops and reallocate to pulses and fruit and vegetables, promote sustainable intensification to increase yields, and consider alcohol, tea and coffee in measures to change food systems. Diversify the types of food produced and agricultural land used.	(Bais-Moleman *et al* 2019; Chen *et al* 2019; Ibarrola-Rivas *et al* 2022; Jha *et al* 2023; Zhu *et al* 2023a)

**Regional approaches to develop food policy:** work with local government to develop place-based policies, based on regional and cultural context, rather than taking a uniform approach. Dietary guidelines should also be based on local food culture.	(Hu *et al* 2022; Stone *et al* 2023; Yin *et al* 2020)

**Foster transdisciplinary collaborations:** develop policies with transdisciplinary stakeholder engagement. In particular, allow citizens to participate in shaping policies.	(Meier *et al* 2014; Stone *et al* 2023)

Recommendations for incorporating sustainability into dietary guidelines were relatively consistent, either suggesting that environmental sustainability in general needs to be considered when developing the guidelines, or specifically that recommendations for meat should be reduced. The recommendation from Candy *et al* (2019, p. 23), summarises the main points arising from those who suggested a holistic approach is needed; ‘Coherent food system-oriented policy approaches are necessary to tackle the social, political and environmental determinants of non-communicable diseases and food insecurity in an equitable manner, and need to be coordinated across multiple sectors and levels of government’ (Candy *et al* 2019). Arrieta and colleagues (2022, p. 8), recommend a framework that recognises the ‘totality of food systems’ and their ‘inter-weaving elements’, to help minimise trade-offs and integrate the health, environmental and social impacts of food production and consumption (Arrieta *et al* 2022).

### 3.5. Availability of data

As mentioned, the percentage changes for each food group were calculated based on the data in each paper, if the results were not already presented in this format. When the relevant data was not available in the paper or supplementary material, authors were contacted to request access.

Approximately twenty-six authors were contacted for this data. However, twelve papers could not be included in the summary of dietary impacts, because underlying data was not readily available, or not available in the format needed for analysis. For example, if all plant-based food was grouped together—fruit, vegetables, carbohydrate and pulses, then it was not suitable for analysis as data could not be categorised into the relevant food groups. The availability of data from previous studies is important as it makes it easier for other researchers to build upon existing analysis, as well as helping to identify or clarify any potential errors.

## 4. Discussion

It is clear from the uptick of relevant papers since 2019 that analysing the land use impacts of dietary shifts towards dietary guidelines is an area of increasing interest, given that the majority (77%) of papers included in this review have been published within the past six years. While a spread of countries was included in these analyses, papers clustered around certain countries, particularly the US and China. However, unlike the review by Webb and colleagues, there were a reasonable number of papers included from low- and middle-income countries (Webb *et al* 2023). The range of countries eligible for this review was limited by the need for dietary scenarios to be based on national dietary guidelines. Although over 100 countries have national dietary guidelines, there are still many countries that do not, particularly lower income countries (FAO 2024).

Many studies focused on the US and China which covered different time periods, and this means that these country analyses were sometimes based on different versions of dietary guidelines, for example the USA published new dietary guidelines in 2015 and 2020. It is important to bear in mind that even when the studies covered the same version of the dietary guidelines, different methods were used, and in LCA studies often different systems boundaries were chosen.

### 4.1. Comments on overall approaches taken

Very few papers used a spatial approach for modelling land use change. Life-cycle analysis was used most often by papers in the sample to assess the land use and environmental impacts of shifting diets to align with dietary guidelines. While this approach is useful in that it can give an estimate of the impacts of diet change on the amount of land use, along with other environmental impacts, it can be difficult to use this approach to assess spatial implications of changes in land use (Chaplin-Kramer *et al* 2017). While the overall quantity of land use change may remain the same as other approaches, the spatial allocation of land use change is important to consider, particularly regarding whether modelled land use changes are actually feasible for the country. The production capability of the land available, as well as navigating conflicts with existing land uses such as urban areas, are key to informing policy and practice decisions about agricultural land use.

Being able to identify where land use changes might happen spatially, also allows for the use of methods such as species distribution modelling to measure impacts on biodiversity and explore how different species might respond to the land use change. The use of spatial data also has its limitations however, notably the variation of results based on resolution of the data. Spatial analysis can also be limited by the type of land data available, for example whether cropland is represented as one land use type, or whether it is possible to access data on the specific foods that are produced on different areas of land.

The lack of standardisation in the approaches and system boundaries used in the LCA studies, made it difficult to meaningfully compare results, as some studies only included environmental impacts up to food leaving the farm, or arriving at the store, while others included all impacts linked with the full life cycle of the diet. In some cases, this was due to a lack of availability of data for certain stages (Meier and Christen 2013), however, in many cases there was no justification given for the selection of the system boundary (Bais-Moleman *et al* 2019, Arrieta *et al* 2022). While the boundaries chosen will depend on the aim of the research and the data available for that region and food/production type, it would be best to standardise terminology for food systems LCA to maximise clarity. Traditionally the boundaries used in LCA are cradle (production), farm gate or store and grave (end of life/disposal) (Cucurachi *et al* 2019). Some other terms included in papers in this review were ‘cradle-to-plate’ and ‘cradle-to-mouth’ and ‘farm-to-fork’, however it is unclear whether these boundaries are the same as ‘cradle-to-grave’. Again, this introduces complications when researchers are looking to compare results across studies. Goossens and Schmidt recommend developing a centralised environmental database for food to be used for a standardised ‘farm-to-fork’ approach to LCA (Goossens and Schmidt 2025).

### 4.2. Impacts on diet and land use

While the exact level of changes varied between papers, all found there would need to be an increase in fruit and vegetables, and all but Ridoutt *et al* indicated a reduction in meat (Ridoutt *et al* 2020, 2021). Not all papers included sugar in their analysis, but for all studies which did, results indicated a reduction in intake was needed. Results for starchy carbohydrates, dairy, fish and oil consumption were more varied.

Regarding the increase in meat found by Ridoutt *et al* one potential contributor to this discrepancy is that the Australian dietary guidelines have higher allowances for meat consumption within the recommendations than some other countries included in the review. The Australian dietary guidelines allow for up to three daily servings of protein, which include red meat, poultry, fish, eggs and legumes, nuts and seeds, and up to 455 g of red meat per week (National Health and Medical Research Council, Department of Health and Ageing, 2013). These dietary guidelines are from 2013, and the Australian Government is currently in the process of updating the guidelines, which are expected to be published in 2026 (National Health and Medical Research Council 2024). While the other analysis focused on Australia did not find there would be an increase in meat consumption, the authors included specific optimisation criteria to reduce consumption of red meat (Candy *et al* 2019). It is also important to note, that both papers by Ridoutt *et al* acknowledge funding from Meat & Livestock Australia (MLA 2025).

There were mixed results when it came to changes in dairy, but the increases seen are in line with work from Springmann et al, which modelled the changes needed to meet dietary guidelines in 85 countries, and found that most countries would require an increase in dairy consumption compared to current diets (Springmann *et al* 2020). The studies with the highest level of increase were focused on China. This may be partly due to the recommendations for dairy consumption increasing from 8% of the diet in 1997%–26% of the diet in 2022 (Zhu *et al* 2023b). Despite these increases, China traditionally has quite a low consumption of dairy (Yang *et al* 2023), due to a high prevalence of lactose intolerance (Yongfa *et al* 1984, Yang *et al* 2013), and a more plant-based diet, although this is now changing (He *et al* 2019, Zhu *et al* 2023b).

Some authors looked at the impacts of cropland and pastureland separately. Ibarrola-Rivas and colleagues suggested this should be done as the impacts can be different, depending on the land use type (Ibarrola-Rivas *et al* 2022). Peters also recommended that changes in cropland and pastureland should be reported separately when modelling the impact of dietary change scenarios on carrying capacity of land, as the impacts on land use can vary widely depending on the composition of the diet (Peters *et al* 2016). This disaggregation would provide a better insight in how dietary changes could impact different types of land use, which is important considering that not all land is suitable for all types of agricultural use.

### 4.3. Inclusion of biodiversity

While many papers included analysis of other environmental impacts in addition to land use, these mostly focused on GHGs and water use. Only four papers included assessments of the impacts this dietary shift could have on biodiversity (Martin and Brandão 2017, Ridoutt *et al* 2020, Springmann *et al* 2020, Read *et al* 2022). This is similar to the results of a review by Steenson and Buttriss looking at the impacts of a broader range of healthy and sustainable diets, which found that only one of the 29 studies looked at the potential impacts on biodiversity (Steenson and Buttriss 2021).

Given the importance of biodiversity to the food system, and the extent to which the food system can affect biodiversity, this aspect of environmental impacts needs to be included more often in research examining the environmental impacts of dietary guidelines. Although impacts on biodiversity were not assessed in most studies, it was promising that many authors recognised that biodiversity does need to be included in this line of research in the future, however there are many barriers to doing so. While most papers did not give a reason for not including biodiversity in their analysis, two cited a lack of adequate data. However, authors did not specify exactly what data would be required. As there are global databases for biodiversity (GBIF: The Global Biodiversity Information Facility 2025), more clarity on exactly what data they are lacking, and what format biodiversity data would need to be in to include in these models, could help future researchers fill those data gaps. Some papers mentioned that land use and eutrophication are good proxies for biodiversity, and many other studies link their results on land use to potential impacts on biodiversity (van Dooren *et al* 2014, van Dooren and Aiking 2016). The risk with this approach, is that different types of land use change may have different impacts (e.g. cropland versus pastureland), a total land use change statistic may not be enough to infer possible impacts on biodiversity.

### 4.4. Data sources

There was often a long period of time between baseline data collection and modelling analysis taking place. This makes it quite likely that the data are no longer entirely representative of national diets, and potentially do not take into account any dietary transitions that have occurred in the intervening time period. He and colleagues (2019) highlighted this gap between their 2011 dietary dataset used for their analysis published in 2019. The authors suggested that, in the context of rapid urbanisation and lifestyle changes in China, the potential to improve diets and policies aiming to do so may have changed during that time (He *et al* 2019). When analysing dietary or food supply change, regularly collecting, up to date data is essential to explore the most pressing changes needed to move towards healthier diets. This is particularly true in recent years after major food system shocks including a global pandemic, cost of living crisis and an increase in dietary change for environmental reasons (Bennett *et al* 2021, Stewart *et al* 2021, Dicken *et al* 2022, Bimbo 2023). National governments should carry out more frequent national dietary surveys to ensure any of these potential changes are captured. The gap between dietary data collected and publication was lowest when using a combination of both national and FAOSTAT data, however, only a very small subset of papers combined both dietary data sources (Zhu *et al* 2023a, 2023b).

While FAOSTAT is updated more frequently than most national dietary assessments, many authors chose to use FAO data from 2010/2011 as the environmental data they used were only available for that time point. However, there are also limitations to FAO dietary data. First, FAO Food Balance sheets do not take into account food loss and waste. This data also may be more representative of the overall food supply, rather than individual consumption. Therefore it depends on the aim of the study, which type of data would be more appropriate. Smith and colleagues identified discrepancies between FAO Food Balance Sheets and national food purchasing surveys in the UK, and suggested that underreporting and food waste were the most likely contributors to these discrepancies (Smith *et al* 2023). An earlier analysis found an overestimation of the quantity of food supply from FAO, particularly linked with imports, when compared to national agriculture and trade data (Kelly *et al* 2025).

### 4.5. Policy recommendations

The review from Webb and colleagues highlighted the lack of papers engaging with policy implications of results (Webb *et al* 2023). In contrast to these previous results, just over half of papers in the current study discussed policy in relation to their results. The most frequent recommendations were to incorporate sustainability into dietary guidelines (*n* = 12), and to have a more holistic approach to food systems policies (*n* = 7), with coordination from different stakeholder groups, to support dietary guidelines and achieve food systems change. Many countries have started updating their national dietary guidelines to incorporate different elements of environmental sustainability (Ministry of Food Agriculture and Fisheries of Denmark Danish Veterinary and Food Administration., 2021; Ministry of Health Brazil 2014, Swedish National Food Agency (Livsmedelsverket) 2015). Aside from recommendations to update dietary guidelines and take more holistic, joined-up approaches to food systems, most recommendations focus on different aspects of production, consumption, or a combination of both. Considering the complexity of both food and land systems, it would be beneficial for more authors to also include recommendations linked with the ‘missing middle’ to help facilitate changes that are needed to both production and consumption of different foods (Veldhuizen *et al* 2020).

Although some authors did make specific policy recommendations, there were many that included a more general discussion of potential policies (*n* = 10). These papers often included a policy implications section, in which authors suggested that their results could be used to inform policy, but they did not always make direct policy recommendations based on their research. Given the links with dietary guidelines, this area of research would benefit from more direct engagement with the specific policy implications of results.

### 4.6. Gaps identified and guidance for future research

Three main gaps observed from conducting this review are: the absence of biodiversity outcomes being measured; lack of transparency around methods and access to data; and variations in the systems boundaries set in LCA and of terms used (for example cradle-to-mouth, cradle-to-plate, cradle-to-grave, farm-to-fork).

Biodiversity was one of the least frequently included environmental outcomes. Food systems researchers should engage more with biodiversity, particularly when analysing the impacts of dietary change. Concerted efforts are needed to identify exactly what data and accessible tools are needed for researchers to more easily include biodiversity in analyses of environmental impacts of dietary change. Additionally, more biodiversity data needs to be collected at a national level.

A major challenge during the course of this review, was identifying exactly what researchers had included in their scenarios. Researchers carrying out scenario modelling should include a table summarising all assumptions and features of dietary scenarios and the indicators used when writing up their findings. Whenever possible, final datasets should be uploaded to a data repository or include in supplementary materials. Better access to data and description of methods would facilitate more transparency and allow future research to build on the existing research base. This supports the observation from Webb *et al* which cited the lack of space authors have when writing up methods as a barrier to more detailed explanations of methods, data sources and explanations about choices of indications (Webb *et al* 2023).

Even when the same overall method was used, the lack of standardisation in terms of functional unit, terminology and system boundaries used meant that results of different studies were not easily comparable. The research community should agree on which system boundary is ideally used to assess dietary impact, and coordinate efforts to collect the data required for all post-farm stages of LCA. Standard terminology and definitions of these boundaries should be used. The variation in LCA systems boundaries has also been highlighted by Goossens and Schmidt, and as they suggest, a standard approach of measuring farm-to-fork impacts of diet shift would improve comparability between studies (Goossens and Schmidt 2025). If this is not possible, for example due to lack of data, using standard terminology to define the systems boundaries used would support comparability of studies. There are also groups, such as Hestia in the UK, which are extending the work of Poore and Nemecek (2018) to help standardise the LCA process for farms, businesses and researchers, and making data on environmental impacts of food supply chains more easily accessible (HESTIA 2025).

Table 4 outlines a set of questions to help guide the development of dietary scenarios and future research on the environmental impacts of dietary change. These questions are informed by some of the gaps and best practices identified from this review. As this review highlights the wide variation in the methods and data used, these questions are intended to help move towards a more transparent process, which is the first step towards consensus on gold standard approaches and better standardisation.

**Table 4.** Questions to guide the development of research analysing impacts of dietary change on land use and other environmental impacts.

Research element	Question	Recommendation
**Design**	How do you define sustainability?	From the outset of the research project, have a clear and agreed definition of sustainability, and include this in the write up of the research. For example, in the context of the research does sustainability only refer to environmental, or also economic sustainability. If only environmental, what aspects are included?
	Are a wide range of environmental factors included in the analysis (particularly biodiversity, eutrophication, energy and fertiliser use, which are less frequently included)	If any of the factors listed are not included, explain why it was not possible to include them, or why they were not considered necessary for the analysis. If not included due to lack of data, describe exactly what data is needed to include this factor in the analysis, including: • what indicators and units are needed • the format the data need to be • software compatibility needed
	What assumptions are made in the overall design of the research and in specific scenarios?	When writing up the research, summarise all assumptions and parameters for all scenarios explicitly in a table
	When carrying out LCA • What system boundary will be used and why? • What functional unit will be used and why? How might the use of this functional unit impact the interpretation of the results?	Explicitly define the system boundary and why it has been selected. Ideally a farm-to-fork approach should be used, but when not possible, explain why and what would be needed to ensure a farm-to-fork boundary could be used for the same analysis in the future.

**Data**	Is the data used a proxy indicator (such as habitat loss for biodiversity), or a direct measure (e.g. species abundance)?	If indicators are used, rather than direct measures, explain why this is the case, and how this may impact the interpretation of the results.
	Is it possible to disaggregate the analysis on environmental impacts (such as cropland and grazing land)?	When possible disaggregate environmental factors such as blue/green water and types of agricultural land use. If not possible, explain what is needed in terms of data updates in software/analysis tools in order to allow for disaggregation.
	Can final datasets and code be made available?	Ideally include data in its final form used for analysis, rather than only the raw data used. This will prevent future researchers needing to duplicate work, and will help support secondary analysis

**Interpretation of results and policy implications**	How feasible are the land use changes with the land available and suitable for farming in the country under study?	Discuss the feasibility of these changes in the discussion, and highlight any data available on the amount of suitable agriculture land available in the country.
	How do the results link with the current policy landscape?	Identify the most relevant policies linked with agriculture of dietary change that may be relevant. It would be particularly helpful to highlight any current policy developments that the study results could be factored in to.
	How might current policies impact the feasibility of the scenarios being implemented?	Discuss whether there are any policy movements which may support or hinder the changes modelled, either in terms of promoting dietary change, or facilitating access to or change in use of agricultural land.
	What policy changes are needed to help facilitate the changes modelled in the scenarios?	Consider what recommendations you would make to policymakers on both a national and local scale, to help achieve the diet and land use changes modelled (if that is a desirable outcome). Alternatively, if results indicated potential negative impacts on some environmental factors when moving towards healthy diets, consider what measures policymakers could put in place to mitigate this.

### 4.7. Strengths and limitations

One of the main strengths of this review is that it has a wide scope of the data captured from papers, including methods, data sources, dietary changes, impacts on land use, GHGs, water, biodiversity, and policy recommendations. This provides an extensive overview of the current state of research in this area, which can inform future researchers conducting similar research on the gaps in the current literature, along with the challenges in the approaches that must be navigated, such as a lack of standardisation. In terms of limitations, the papers included in the review were restricted to those written in English, and as the focus was only on analysis including national dietary guidelines, thus excluding countries without dietary guidelines. Furthermore, due to the range in approaches and assumptions used in scenarios, it is not possible to perform statistical analysis of the environmental impacts of aligning diets and food supplies with dietary guidelines. As this area of research develops, and approaches become more standardised, it may be possible to do this type of analysis.

While this review focuses on what the potential impacts could be if current diets were fully aligned with national dietary guidelines, it would also be important to understand what the impacts are, if any, on both the diet and the environment when national dietary guidelines are updated, or introduced for the first time.

## 5. Conclusions

The changes needed to align diets with national dietary guidelines are relatively consistent across studies and regions, with most needing to increase fruit and vegetables, and reduce meat and sugar. The environmental impacts of this change in diets and food supply, however, are mixed, highlighting the need to include multiple environmental factors in diet and land use scenarios, to get a broader picture of the impacts and potential trade-offs or co-benefits, so these can be managed effectively. While many studies do also include the impacts on GHGs and water use, very few include biodiversity when modelling the impacts of aligning diets with national dietary guidelines. Considering the importance of biodiversity to the food system, it is essential that it is included in more studies measuring the land use impacts of diets aligned with dietary guidelines.

This area of research is likely to continue to grow in the coming years and the clear reporting of scenarios, assumptions and easy availability of datasets used and created will make it easier for future researchers to build on the existing research. There is also scope for deeper engagement in the policy implications of this area of research, with more emphasis placed on the actionable policy recommendations that can be developed from these studies.

## Data Availability

The data that support the findings of this study will be openly available after acceptance (but before publication) at the following URL/DOI: https://figshare.com/authors/Niamh_Kelly/20500568. Supplementary data 1 available at: https://doi.org/10.1088/2976-601X/ae7089/data1.
